# High Warming Restricts the Growth and Movement of a Larval Chinese Critically Endangered Relict Newt

**DOI:** 10.3390/biology14080942

**Published:** 2025-07-27

**Authors:** Wei Li, Shiyan Feng, Shanshan Zhao, Di An, Jindi Mao, Xiao Song, Wei Zhang, Aichun Xu

**Affiliations:** 1 College of Life Sciences, China Jiliang University, Hangzhou 310018, China; lw0512@cjlu.edu.cn (W.L.); fsy0714@cjlu.edu.cn (S.F.); echoannnndy@gmail.com (D.A.); xsong@cjlu.edu.cn (X.S.); springlover@cjlu.edu.cn (A.X.); 2 Keqiao Branch of Shaoxing Ecology and Environment Bureau, Shaoxing 312030, China; polaris_nuan@163.com; 3 Natural History Research Centre, Shanghai Natural History Museum, Shanghai Science & Technology Museum, Shanghai 200041, China

**Keywords:** climate change, basal metabolic rate, locomotor performance, life history trait, amphibia, *Echinotriton chinhaiensis*

## Abstract

Amphibians are the most threatened vertebrates, yet their resilience in relation to growth and locomotor performance with rising temperatures remains poorly understood. Here, we chose a critically endangered amphibian—the Chinhai spiny newt (*Echinotriton chinhaiensis*)—as the study species and set four water temperature gradients (20 °C, 24 °C, 28 °C, and 32 °C) to simulate climate changes. The thermal performance to climate warming was quantified by measuring morphometric parameters, basal metabolic rate (oxygen consumption rate), and the locomotor performance of Chinhai spiny newt larvae. We found that the optimal temperature range for Chinhai spiny newt larvae is 24–28 °C. Within the temperature range of 24–28 °C, the growth, oxygen consumption rate, and locomotor performance of the larvae were positively correlated with temperature. High temperatures inhibited larval growth, oxygen consumption rate, and locomotor performance, and the temperature threshold was 32 °C. In addition, Chinhai spiny newt larvae are more sensitive to acute temperature changes, meaning that climate-driven extreme events (e.g., heatwaves and droughts) pose significant threats to their larvae. The optimal temperature range obtained from this study could guide artificial breeding and early warming; future studies should integrate controlled temperature fluctuations in order to understand the thermal adaption of this threatened species.

## Simple Summary

Knowledge of amphibian thermal response mechanisms is critical in order to assess their vulnerability to climate warming. This study quantified the effects of different constant acclimation temperatures on the growth, basal metabolic rate, and locomotor performance of Chinhai spiny newt larvae. The results showed that the optimum temperature range for Chinhai spiny newt larvae was 24–28 °C, and the temperature threshold was 32 °C. Low temperatures inhibited larval growth and basal metabolic rate, but increased locomotor performance. On the other hand, high temperatures restricted larval growth and decreased basal metabolic rate, ultimately affecting their locomotor performance. In addition, acute temperature changes significantly affected the basal metabolic rate and impaired locomotor performance of the larvae. The optimal temperature range obtained from this study could guide artificial breeding and early warming.

## 1. Introduction

Climate warming exerts multifaceted ecological impacts through two primary pathways. Firstly, climate warming increases the prevalence of extreme weather events and changes rainfall patterns, which can damage both the regional and global environment; these combined changes disrupt phenological synchrony across ecosystems [1]. Secondly, rising temperatures directly affect the development of wildlife by speeding up life cycles and shortening breeding periods [1]. Amphibians demonstrate heightened vulnerability to these thermal perturbations due to their ectothermic physiology and complex life history traits [2]. Therefore, elucidating the adaptive mechanisms of amphibians to shifting thermal regimes constitutes a critical priority for developing evidence-based conservation frameworks.

Phenotypic plasticity is a method by which animals may moderate the impact of climate variability through adaptive flexibility. Phenotypic plasticity is a critical adaptive mechanism that can be used to maintain survival and reproductive fitness when exposed to environmental stressors [3]. The plasticity of amphibians facilitates dynamic adjustments in morphological architecture (e.g., body proportion scaling) and physiological thresholds (e.g., metabolic rate modulation) when confronting thermal extremes or habitat alterations [4]. Temperature changes trigger distinct developmental responses in amphibian larvae [5,6]. Studies have shown that some tadpoles demonstrate accelerated somatic cell growth in response to elevated temperatures, which is a phenomenon of thermoregulatory adaptation [7,8]. This growth acceleration precipitates the premature initiation of metamorphic climax, curtailing the larval developmental stage. Metabolic trade-offs ultimately result in a reduced post-metamorphic body size [5]. An amphibian’s physiological performance is modulated by both natural seasonal thermal fluctuations and anthropogenic thermal acclimation regimes [9]. This aligns with the “beneficial acclimation hypothesis” (BAH), which posits that prolonged environmental conditioning enhances organismal fitness within corresponding habitats [10]. However, BAH applicability remains contentious, reflecting constrained resource allocation strategies and evolutionary trade-offs. Amphibians demonstrate an adaptive capacity through both short-term phenotypic adjustments and long-term thermal acclimation processes [11]. Understanding the interplay between synergistic and antagonistic mechanisms is crucial for predicting physiological thresholds and assessing the vulnerability and adaptive capacity of amphibians under the conditions of climate change [12].

Climate warming and the subsequent rise in water temperatures have significant impacts on amphibians, especially species that are highly depend on seasonal ponds (such as temporary ponds and stream shallows) for reproduction and larval development [13]. As ectotherms with limited mobility, individuals in the aquatic larval stage are especially sensitive to water temperature changes [2]. Therefore, it is crucial to quantify the physiological and ecological adaptation characteristics (such as the phenotypic plasticity) of amphibians and determine their optimal thermal ranges. Current studies primarily employ constant and fluctuating temperature gradients to simulate climate warming in order to explore thermal adaptation strategies [14,15]. Among them, the constant temperature design is a traditional and classic research method that helps to establish the basic temperature–response relationship and contribute to artificial breeding conditions [16]. The fluctuating temperature design is usually conducted based on the results of the constant temperature design in order to simulate diurnal and seasonal temperature variation patterns under climate warming scenarios [14,17].

Climate warming drives significant alterations in amphibian life-history strategies [18]. These changes are related to the survival and reproduction of amphibians [13]. According to the temperature–size rule, elevated ambient temperatures drive faster larval growth under thermal stress, but lead to a reduced body size post-metamorphosis [19,20,21]. Empirical validation comes from plateau brown frog (*Rana kukunoris*) tadpole studies, where warming triggered earlier metamorphosis. These life-history strategies caused significantly smaller body sizes at forelimb emergence and tail resorption compared to the control groups [22]. As a key determinant of fitness, body size shapes critical ecological interactions, such as predator–prey dynamics, reproductive success, and resource competition [23]. Reductions in body size may increase extinction risk by impairing these functions.

The basal metabolic rate (BMR) represents the energy expenditure required for homeostatic maintenance in resting organisms and serves as a key indicator of metabolic activity [24]. In amphibians, BMR shows a high thermal sensitivity owing to ectothermy and complex life-history traits [25]. Rising temperatures due to climate change increase metabolic demands in amphibians, which alters the balance of energy allocation among growth, the basal metabolic rate, and locomotor performance [26]. This involves the metabolic theory of ecology, which posits that organismal metabolic rates scale exponentially with ambient temperature [27]. Eastern red-backed salamanders (*Plethodon cinereus*) displayed an elevated BMR under hyperthermic conditions through upregulated mitochondrial respiration [28]. Tadpoles of the African clawed frog (*Xenopus laevis*) exposed to elevated water temperatures exhibited a reduced thermal tolerance and an increase in BMR relative to control groups [29]. These findings collectively demonstrate that rising temperatures directly increase the BMR of amphibians, necessitating greater energy expenditure for basic physiological maintenance.

Locomotor performance (e.g., predation, antipredation, and migration) constitutes a vital factor influencing amphibian survival and reproductive success [30]. Climate warming impairs amphibian locomotor performance by disrupting the thermal–metabolic balance, reducing muscle contractile efficiency and altering energy allocation [31]. These physiological disruptions compromise individual fitness and destabilize ecosystems through shifts in species competition and cross-trophic energy flow [32]. Elevated temperatures induce significant alterations in amphibian locomotor performance [33]. For instance, tadpoles of the African clawed frog from high-elevation cold habitats exhibit a reduced locomotor capacity compared to lowland conspecifics in warmer environments [34]. Similarly, elevated temperatures enhance both thermal tolerance thresholds and burst swimming capacity in adult *Nanorana pleskei* [35]. In contrast, cool-adapted common frogs (*Rana temporaria*) achieve peak jump kinematics at 20 °C, outperforming warm-adapted frogs whose optimal performance occurs at 25 °C [36]. Collectively, these results demonstrate that rising temperatures directly modulate amphibian locomotor performance, with cascading effects on individual survival and reproductive fitness.

The Chinhai spiny newt (*Echinotriton chinhaiensis*), a urodele amphibian that is endemic to China, belongs to the Salamandridae family; they represent one of the most ancient extant lineages of salamanders. This species is listed as “Critically Endangered” on the IUCN Red List [37], and is narrowly distributed in Ningbo, China. The Chinhai spiny newt undergoes a quadripartite developmental process, consisting of embryonic development, larval development (subdivided into the hind limb three-toed stage, four-toed stage, five-toed stage, and external gill regressing stage), subadult development, and adult maturation [38]. The larvae are highly dependent on a specific type of small wetland—still-water ponds. The reason for the small population in the wild is partially attributed to metamorphic failure. On the one hand, the low hatching success could be due to suboptimal climatic conditions and oviposition site suitability [39]. On the other hand, the mortality of larvae in still-water ponds could be driven by habitat degradation and resource limitation, which exacerbates predation pressure and intraspecific cannibalism. Climate warming exacerbates these threats through the accelerated desiccation of still-water ponds. Rising water temperatures coupled with declines in water levels critically compromise larval viability in these thermally vulnerable microhabitats [40].

This study simulated climate warming scenarios through thermal acclimation gradients and acute temperature change experiments. We specifically quantified larval responses in growth, basal metabolic rate (oxygen consumption rate), and locomotor performance. These indexes reflect individual fitness optimization under climate-mediated environmental stochasticity. In particular, we aimed to determine (1) the impacts of changes in acclimation temperatures on morphometric parameters (body mass, snout–vent length, head width, and head length); (2) the effects of acclimation temperatures and acute temperature changes on basal metabolic rate through oxygen consumption dynamics; and (3) the effects of acclimation temperatures and acute temperature changes on locomotor performance. We predicted that (1) both low and high temperatures inhibit larval growth, while specific temperature ranges promote larval growth; (2) low and high temperatures inhibit larval locomotor performance, but high temperatures increase larval basal metabolic rate; and (3) larvae would exhibit changes in basal metabolic rate and locomotor performance in response to temperature changes and/or acclimation temperature changes. These findings could reveal the response of Chinhai spiny newt larvae to climate changes in relation to growth, basal metabolic rate, and locomotor performance, as well as guiding habitat conservation and monitoring in the context of climate warming.

## 2. Materials and Methods

### 2.1. Study Species and Husbandry

**Figure 1:**
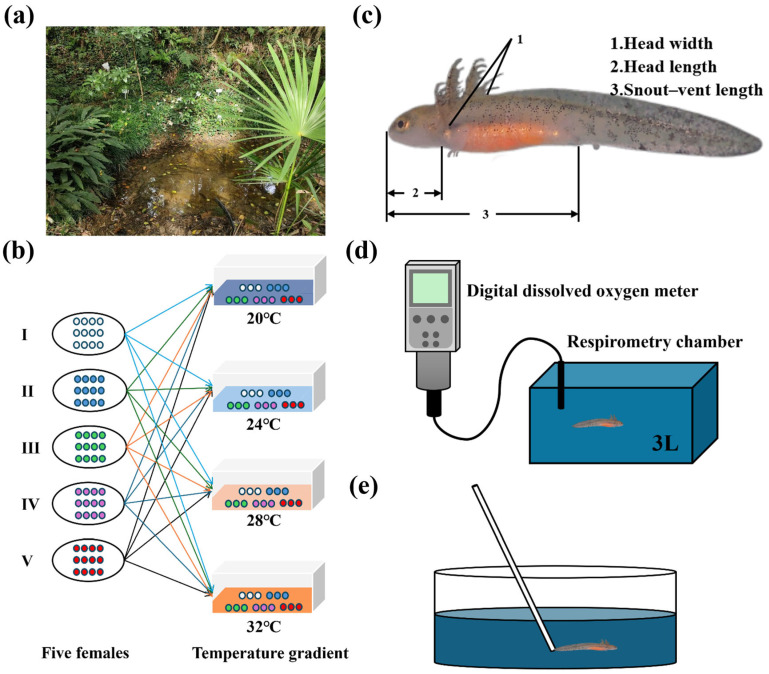
Stages of experimental design from collection to experimental set up. (**a**) Nests collected from natural still-water ponds. (**b**) One repetition group. Larvae were randomly selected from each female and transferred to four temperature groups (acclimation). Upon reaching the hind limb five-toed development stage, these larvae were then redistributed across all four temperature groups (experiment). (**c**) Morphometric parameter measurement. (**d**) Basal metabolic rate measurement. (**e**) Locomotory performance measurement.

Along with artificial breeding project of the Chinhai spiny newt, we collected Chinhai spiny newt egg masses from April to May 2024, with permission from the Ningbo Bureau of Natural Resources and Planning (No. NBDW-20221082) [40] and the China Jiliang University (No. 2023039) [41]. The collection was carried out in the Chinhai spiny newt Protected Zone (29°48′24″ N, 121°51′12″ E), which is located in Ningbo, Eastern China. All egg masses came from the banks of four breeding still-water ponds in the same reserve (Figure 1a). All the selected eggs exhibited identical developmental stages, as determined through embryonic morphological criteria [42]. These eggs were also transported to the Chinhai spiny newt Artificial Breeding Laboratory located in the Chinhai spiny newt Protected Zone. The eggs were allocated to cylindrical incubation chambers (diameter = 25.0 cm; height = 7.5 cm). Each chamber contained a hydrophilic porous material (length = 17.0 cm; width = 12.0 cm; height = 2.0 cm) for moisture regulation. The water depth was maintained at 1.5 cm through automated replenishment, ensuring the complete saturation of the porous material in order to sustain developmental humidity. Following hatching success, Chinhai spiny newt larvae were systematically transferred to rectangular rearing tanks (length = 45.0 cm; width = 29.5 cm; height = 14.5 cm). These rearing tanks maintained a water column depth of 4.5 cm.

### 2.2. Experiment Design

Given the extreme endangerment of the Chinhai spiny newt, this study rigorously adhered to the 3R principles (Replacement, Reduction, Refinement) [43] throughout its experimental procedures. In this study, we investigated the response of Chinhai spiny newt larvae to climate change in terms of morphometric parameters, basal metabolic rate, and locomotor performance. Morphometric parameters refer to body weight, snout–vent length, head length, and head width; basal metabolic rate refers to oxygen consumption rate. Meanwhile, we set up the different temperature gradients to investigate the growth, metabolism, and exercise response strategies of the Chinhai spiny newt. Following the completion of all experimental procedures, we conducted field acclimatization and released all the individuals into their natural habitats.

Current studies exploring the thermal response to warming involve both the constant temperature design [15,16,44] and the fluctuating temperature design [14,17,45,46]. The constant temperature design is a classic and widely used methodology in thermal adaptation research [15,16,44]. By subjecting the study species to constant temperature gradients, the species’ physiological and ecological responses are quantified. The fluctuating temperature design is a method that is used to simulate the diurnal and seasonal temperature change patterns under climate warming, exploring the response of physiological functions, behavioral strategies, population dynamics, and ecological adaptation [14,17,45,46]. Since the Chinhai spiny newt is a restricted-range species, regional temperatures are relatively homogeneous. Due to the limitations of the experimental equipment, we employed the constant temperature design in the current study.

In this study, water temperature involves the acclimation temperature and experimental temperature. Firstly, we chose four acclimation temperatures (20 °C, 24 °C, 28 °C, and 32 °C) to quantify the morphometric parameters, basal metabolic rate, and locomotor performance in order to explore the effects of climate warming changes. Chinhai spiny newt larvae were maintained at a constant temperature until they reached the hind limb five-toed developmental stage. We measured the morphometric parameters of all experimental individuals (240 larvae) twice a week during the temperature acclimation period. Then, the 128 larvae that reached the hind limb five-toed developmental stage were exposed to four experimental temperature conditions (20 °C, 24 °C, 28 °C, and 32 °C) for the measurement of basal metabolic rate and locomotor performance in order to investigate the effects of acute temperature changes in these properties on the larvae. According to the long-term temperature monitoring of still-water ponds in the Chinhai spiny newt Protected Zone, the lowest water temperature in the still-water ponds in wild Chinhai spiny newt habitats was about 22 °C from May to July, while the highest water temperature was about 27 °C [40] (Figure S1). High temperature stress pre-experiment in the laboratory showed that the maximum tolerance temperature of Chinhai spiny newt larvae ranged from 33 to 36 °C [40]. Therefore, the acclimation temperatures were set as 20 °C in the low-temperature group, 24 °C in the control group, and 28 °C and 32 °C in the high-temperature groups. The experimental design included four constant-temperature treatments (20 °C, 24 °C, 28 °C, and 32 °C), each replicated independently four times (*n* = 4 per treatment).

Because our previous results showed that the optimal rearing density was 15 individuals in each tank [47], we used 15 larvae in each temperature treatment for each repetition. Due to the four repetitions, a total of 240 larvae (15 larvae × 4 temperature treatments × 4 repetitions) that entered the water on the same day from the artificial breeding individuals were used in this study. To control for potential maternal effects, larvae from the same female were distributed systematically in the four temperature treatments in each repetition. Meanwhile, the minimum repetition number (three larvae) needed to be met; therefore, we used 60 larvae from 5 females (12 larvae from each female) in each repetition (Figure 1b). Figure 1b represents one repetition group. Each temperature acclimation group (four replicates in each temperature acclimation group—20 °C: 4 × 15 larvae; 24 °C: 4 × 15 larvae; 28 °C: 4 × 15 larvae; 32 °C: 4 × 15 larvae) was acclimated in intelligent artificial climate boxes (Ningbo Jiangnan Instrument Factoru, Ninbo, China, RXZ-type).

### 2.3. Morphometric Parameters

The morphometric parameters (including larval body weight, snout–vent length, head length, and head width) of all the experimental individuals (240 larvae) were measured. We defined the snout–vent length as the length from the snout end to the cloaca; head length was defined as the snout end to the external gills; and head width was defined as being between the ear posterior edges [48,49] (Figure 1c). We measured the morphometric parameters of all experimental individuals (240 larvae) twice a week during the temperature acclimation period until the Chinhai spiny newt larvae metamorphosed and moved to land. For measurement, each individual was photographed using a digital camera (Shenzhen Zhongwei Kechuang Technology Co., Ltd., Shenzhen, China ZW-U500) controlled by S-EYE software (version 1.4.4.5). A 0.5 cm scale bar was attached to the photographs for length calibration. At the end of the assay, the lengths were measured from the photographs using Image J software (version 1.53, nearest 0.001 cm). Larvae were weighed using an electronic balance (G&G Measurement Plant, Changshu, China, JJ224BF; range: 0–220 g; nearest = 0.0001 g) after surface moisture absorption with paper towels. All individuals were returned to their original rearing boxes after measurement.

### 2.4. Basal Metabolic Rate

The basal metabolic rate (BMR), which reflects the cost of maintaining homeostasis, is commonly defined as the standard metabolic rate (SMR) and is measured as the minimal oxygen consumption in an individual’s resting post-absorptive state [50]. Following the study of Padilla et al. (2024) [26], we used respiratory metabolism analysis to quantify the BMR of juvenile individual Chinhai spiny newts; this was standardized as the mass-specific oxygen consumption and was measured under controlled resting conditions at the species’ ecologically relevant temperature. The basal metabolic rate was measured at the hind limb five-toe stage in larvae. Oxygen consumption rates were determined via closed-system hydrostatic respirometry [51], which is a classic method that quantifies the changes in oxygen consumption. The experimental respirometry system comprised two core components—a 3 L respirometry chamber and a digital dissolved oxygen meter (Figure 1d).

To maintain a relatively consistent growth stage for the experimental larvae, we selected 128 larvae (32 per temperature group × 4 groups) that had reached the hind limb five-toe stage from the initial cohort of 240 larvae for BMR quantification. Each individual was exposed to all four acclimation temperatures for the determination of their mass-specific oxygen consumption rates. A 24 h recovery interval was maintained between successive temperature exposures in order to eliminate residual metabolic effects. Prior to the metabolic measurements, the dissolved oxygen meter probe underwent mandatory two-point calibration. To avoid irreversible physiological stress or metabolic memory effects on larvae, which could be caused by successive temperature changes, we employed randomized sequential measurements while ensuring that a ≥24 h recovery period was allowed after each temperature treatment [35].

Following the study of Lin et al. (2024) [51], the body weight of the Chinhai spiny newt larvae was measured; this was carried out in the same way as in the morphometric parameters described above. Then, the larvae were placed in the respirometry chamber. After 10 min of acclimatization, the dissolved oxygen content of the water in the respirometry chamber was measured. The duration of the experiment was 3 h. At the end of the experiment, the dissolved oxygen content of the water in the respirometry chamber was measured again. During the experiment, all larvae used for BMR measurement returned to normal within 30 min. Finally, the dissolved oxygen difference and the oxygen consumption rate of the larvae were calculated. The oxygen consumption rate can be calculated using the following formula:

$$OR=\frac{\left(DO_{0}-DO_{t}\right)\times v}{w\times t}$$

where *DO*_0_ and *DO_t_* represent the dissolved oxygen content of the water in the respiration chamber of the blank group and the dissolved oxygen content of the water in the respiration chamber of the experimental group (mg·L^−1^); *v* is the volume of the water in the respiration chamber (L); *w* is the body weight of the experimental larvae (g); and *t* is the time of the experiment (h).

### 2.5. Locomotor Performance

Locomotor performance usually refers to the ability of wildlife to accomplish tasks related to survival or reproduction (e.g., predation, antipredation, and migration) through exercise in a given environment. In amphibians, it is specifically reflected in the individual’s sprint speed, exercise duration, locomotor coordination, etc. In the current study, locomotor performance refers to swimming duration until immobility under conditions of constant external stimulation [52]. The locomotor performance was measured at the hind limb five-toed stage in larvae. In order to avoid interference, the locomotor performance was evaluated 72 h after BMR measurements. We used a transparent cylindrical arena with precise dimensions (diameter = 25.0 cm; height = 7.5 cm) to assess the locomotor performance. The containers were placed in a thermostatic climate chamber to ensure that the temperature of the experiment was stable.

As was the case with the BMR measurements, we selected the same 128 larvae (32 per temperature group × 4 groups) for the measurement of locomotor performance. Each individual was sequentially exposed to all four experimental temperatures during testing. A 24 h test interval was systematically maintained between temperature transitions. Following the study of Moore et al. (2025) [53], Chinhai spiny newt larvae were placed in cylindrical containers with a water depth of 4.5 cm at the beginning of the experiment. One larva was placed in each container and the experiment was started after the larvae had been acclimatized for 10 min. A glass rod was used to initiate locomotion, simultaneously triggering chronometric measurement. Upon the cessation of swimming activity, immediate re-stimulation was administered to the posterior region (Figure 1e). This stimulus–response cycle persisted until the point of complete locomotor fatigue, which is defined as >3 s of immobility; at this point, temporal recording ceased. During the experiment, all larvae used for locomotor performance measurement returned to normal within 30 min.

### 2.6. Data Analysis

Linear mixed-effects models (LMMs) were used to analyze the differences in the morphometric parameters (body weight, snout–vent length, head width, and head length) of the Chinhai spiny newt larvae in different water temperatures due to the normality of four morphometric parameters (Figure S2). Considering that the larval body weight reflects the body’s food and energy storage, the snout–vent length reflects larval body size, and the head morphology and length/width correlates with feeding efficiency [8], we analyzed each of these four traits using LMMs to reveal the effects of temperature on the different functional axes of the larvae. We used each morphometric parameter as each dependent variable, while acclimation temperature, acclimation time, and the interaction between acclimation temperature and acclimation time were set as fixed effects. The repetition number was set as the random effect. We created four LMMs with a Gaussian error distribution (one morphological parameter and one LMM) [47]. The fit of the four models fit was tested using the qqnorm function [54]. We applied the ANOVA function with Type III analysis of variance to assess the significance of the main effects and interactions [54]. In addition, we conducted pairwise comparisons with false discovery rate (FDR) correction to characterize time-dependent variations in temperature effects based on the emmeans package (version 1.11.1) [55]. LMMs were created based on the “lme4” (version 1.1.37) package [56].

Generalized linear mixed models (GLMMs) in the glmmTMB package (version 1.1.11) [57] were used to assess the differences in basal metabolic rate and locomotor performance between the different water temperatures. Given the continuous and right-skewed nature of the basal metabolic rate data (Figure S3), we employed a Gamma distribution family [58] when creating the GLMMs. To address the observed overdispersion in the locomotor performance data, we employed a negative binomial distribution family [59] when creating the GLMMs for locomotor performance (Table S1). We chose the basal metabolic rate and locomotor performance as the dependent variables; the acclimation temperature, experimental temperature, and the interaction between acclimation and experimental temperature were chosen as the fixed effects. We included the repetition number as the random factor in order to account for within-treatment variance. Additionally, since body size could influence the basal metabolic rate [60], we incorporated body weight as a random factor in order to account for its confounding effects on basal metabolic rate. The DHARMa package (version 0.4.7) [61] was used to evaluate the model’s fit. The significance of the model was assessed using the ANOVA function, while a Type III sum of squares calculation was applied to test the interaction terms. The emmeans package [55] was also used to calculate marginal means and perform multiple comparisons in relation to the oxygen consumption rate and locomotor performance at different temperature combinations.

All statistical analyses were performed in R version 4.5.0 [62].

## 3. Results

### 3.1. Morphometric Parameters

**Figure 2:**
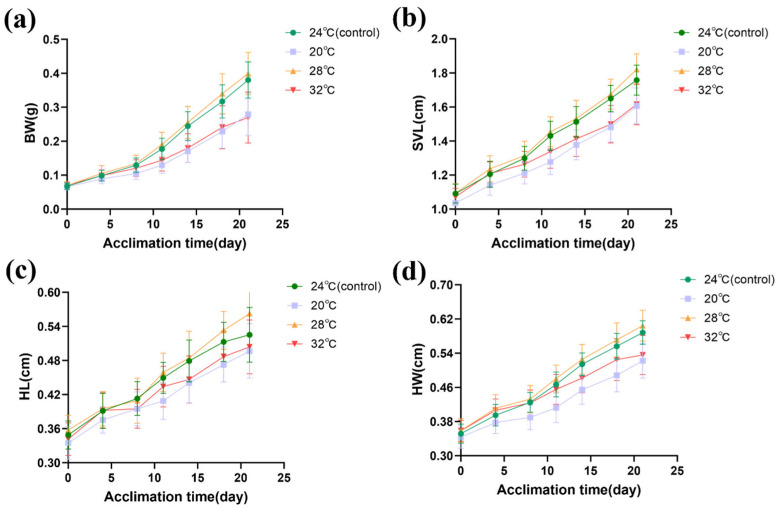
Effects of different treatment temperatures on Chinhai spiny newt larvae in terms of (**a**) body weight (BW), (**b**) snout–vent length (SVL), (**c**) head length (HL), and (**d**) head width (HW).

Acclimation temperatures, acclimation times, and the interaction of acclimation temperatures and acclimation times had significant effects on the morphometric parameters (body weight, snout–vent length, head length, and head width) in Chinhai spiny newt larvae (Table 1). The QQ plot results of the four LMMs show that the residuals effectively conform to the normal distribution; therefore, the model hypothesis holds (Figure S4). Body weight suppression emerged after 8 days of acclimation at 20 °C and after 11 days at 32 °C (Table S2). Post hoc comparisons further demonstrated that the larvae that acclimated at 20 °C exhibited a lower body weight compared to the control group (24 °C) by the 4th day (estimate = 0.084; *t* = 2.579; *p* = 0.026). A similar suppression appeared at 32 °C by the 11th day (estimate = 0.103; *t* = 3.033; *p* = 0.005; Table S3). At 24 °C and 28 °C, the body weights of the Chinhai spiny newt larvae were larger. However, at 20 °C and 32 °C, the body weights were smaller. From 0 to 18 days, the larvae at 20 °C had the smallest body weights; however, from 18 to 21 days, the larvae at 32 °C had the smallest body weights (Figure 2a).

Snout–vent length suppression emerged at both 20 °C and 32 °C after 11 days of acclimation (Table S4). Post hoc comparisons further demonstrated that the snout–vent length of larvae at 20 °C was significantly lower than that of the control group by the 4th day (estimate = 0.098; *t* = 3.797; *p* = 0.002). A similar suppression was observed at 32 °C by the 11th day (estimate = 0.063; *t* = 2.331; *p* = 0.033; Table S5). The snout–vent length of the larvae at 24 °C and 28 °C was larger than that at 20 °C and 32 °C (Figure 2b).

Head length suppression emerged at 20 °C after 11 days of acclimation (Table S6). The head length of larvae at 20 °C was significantly lower than that of the control group by the 11th day (estimate = 0.043; *t* = 5.230; *p* < 0.001). A similar suppression was observed at 32 °C by the 14th day (estimate = 0.030; *t* = 3.656; *p* < 0.001; Table S7). The head length of the larvae was larger at 24 °C and 28 °C, but was smaller at 20 °C and 32 °C. The head length of the larvae at 20 °C was the smallest (Figure 2c).

Head width suppression emerged after 8 days of acclimation at 20 °C and after 14 days at 32 °C (Table S8). The head width of the larvae at 20 °C was significantly smaller than that of 24 °C by the 4th day (estimate = 0.023; *t* = 2.816; *p* = 0.014). The head width of the larvae at 32 °C was significantly smaller than that of the larvae at 24 °C by the 14th day (estimate = 0.025; *t* = 2.928; *p* = 0.006; Table S9). Additionally, the head width of the larvae increased slowly at 32 °C, which suggests that significant suppression appeared in the groups at 32 °C (Figure 2d).

### 3.2. Basal Metabolic Rate

The residual diagnosis results indicated that the GLMMs provided a good fit (Tables S10 and S11). The experimental temperature had a significant effect on the oxygen consumption rate of the larvae (Table 2). Meanwhile, the acclimation temperature and the interaction of acclimation and experimental temperatures had no significant effects on the oxygen consumption rate of the larvae (Table 2). Specifically, when the experimental temperature and acclimation temperature were set at either 20 °C or 32 °C, the larval oxygen consumption rates showed substantial reductions. In contrast, the interaction effects were less pronounced at intermediate temperatures (24 °C and 28 °C).

**Figure 3:**
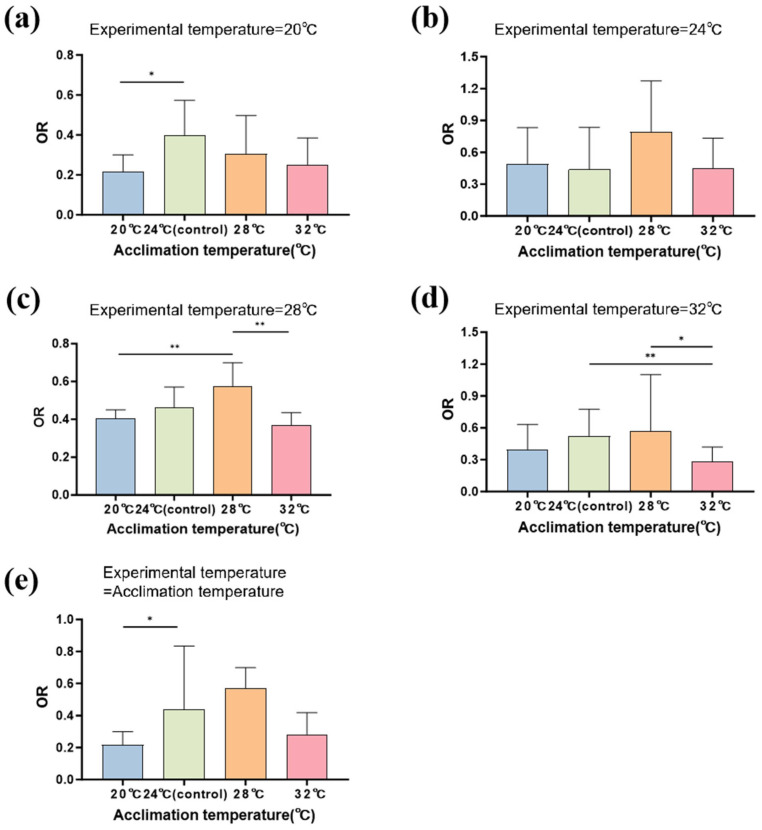
Effects of different acclimation temperatures on Chinhai spiny newt larvae in terms of oxygen consumption rate under different experimental temperatures. (**a**) The experimental temperature was 20 °C. (**b**) The experimental temperature was 24 °C. (**c**) The experimental temperature was 28 °C. (**d**) The experimental temperature was 32 °C. (**e**) The experimental temperature was equal to the acclimation temperature. * represents 0.01 < *p* < 0.05; ** represents 0.001 < *p* < 0.01.

Under the acclimation temperatures, the oxygen consumption rate was significantly lower at 20 °C than at 24 °C (estimate = −0.675; *z* = −2.118; *p* = 0.034; Figure 3e; Table S12). At the 20 °C experimental temperature, the oxygen consumption rate was significantly lower in the larvae acclimated at 20 °C than those acclimated at 24 °C (24 °C: estimate = 0.612; *z* = 2.456; *p* = 0.014; Table S13). At the experimental temperature of 28 °C, the oxygen consumption rate was significantly higher in the larvae acclimated at 28 °C than those acclimated at 20 °C and 32 °C (20 °C: *z* = −2.765, *p* = 0.017; 32 °C: *z* = 3.571, *p* = 0.002; Table S14). At the 32 °C experimental temperature, the oxygen consumption rate was significantly lower in the larvae acclimated at 32 °C than those acclimated at 28 °C and 24 °C (24 °C: estimate = 0.689, *z* = 2.811, *p* = 0.005; 28 °C: estimate = 0.618, *z* = 2.523, *p* = 0.012; Figure 3a–d; Table S13).

### 3.3. Locomotor Performance

**Figure 4:**
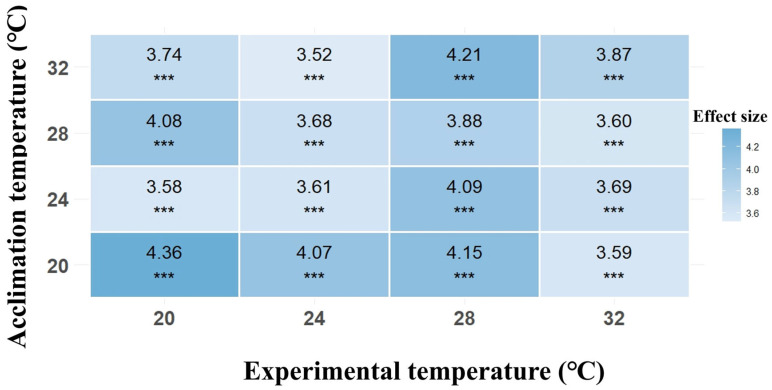
Heatmap of acclimation temperature–experimental temperature interaction effects on locomotor performance. *** represents *p* < 0.001.

The residual diagnosis results indicated that the GLMMs were a good fit (Tables S15 and S16). Both acclimation temperatures and experimental temperatures, as well as their interaction, had significant effects on the locomotor performance of Chinhai spiny newt larvae (Table 3). The analysis of the acclimation–experiment temperature interactions revealed a significant enhancement of the larval locomotor performance when the acclimation and experimental temperatures were maintained at 20 °C or 28 °C. In contrast, these interactive effects diminished at intermediate (24 °C) and high (32 °C) temperature combinations (Figure 4).

**Figure 5:**
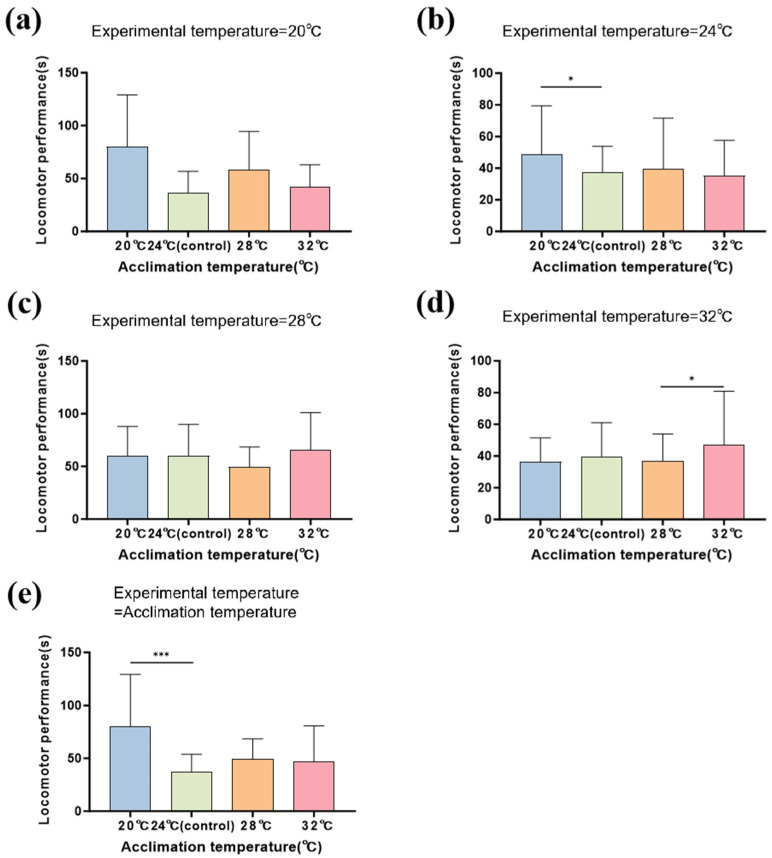
Effects of different acclimation temperatures on Chinhai spiny newt larvae in terms of locomotor performance under different experimental temperatures. (**a**) The experimental temperature was 20 °C. (**b**) The experimental temperature was 24 °C. (**c**) The experimental temperature was 28 °C. (**d**) The experimental temperature was 32 °C. (**e**) The experimental temperature was equal to the acclimation temperature. * represents 0.01 < *p* < 0.05; *** represents *p* < 0.001.

Under the acclimation temperatures, the locomotor performance was significantly enhanced in the larvae acclimated at 20 °C compared to those acclimated at 24 °C (estimate = −0.443; *z* = −3.560; *p* < 0.001; Figure 5; Table S17). Under the 24 °C experimental temperatures, the locomotor performance was significantly enhanced in the larvae acclimated at 20 °C compared to those acclimated at 24 °C (estimate = −0.327; *z* = −2.250; *p* = 0.025). The results at 32 °C showed that the locomotor performance was significantly impaired in the larvae acclimated at 28 °C compared to those acclimated at 32 °C (estimate = 0.272; *z* = 2.240; *p* = 0.025; Figure 5a–d; Table S18).

## 4. Discussion

Amphibians are the vertebrate group that is most vulnerable to climate change [63]. Their thermal adaptation capacities remain insufficiently characterized due to their unique thermal tolerance across geographic distributions and taxonomic lineages [64,65,66]. These variations hinder accurate vulnerability assessments under global climate scenarios [67]. The Chinhai spiny newt is a critically endangered amphibian, whose adaptive mechanism in response to global warming is unclear. This study elucidates the effects of water temperatures on growth plasticity, metabolic regulation, and locomotor performance in Chinhai spiny newt larvae. The basal metabolic rate and locomotor performance exhibit intrinsic physiological correlations in wildlife [68]. Both low (20 °C) and high (32 °C) temperature conditions constrained larval growth and basal metabolic rate; however, low temperatures enhance larval locomotor performance and high temperatures attenuate it. Within a certain temperature range (24–28 °C), larval growth showed positive correlations with temperature elevation. Concurrently, both the basal metabolic rate and locomotor performance exhibited temperature-dependent increases across this thermal gradient. However, when the acclimation temperature reaches its temperature threshold (32 °C), the growth, basal metabolic rate, and locomotor performance of the larvae decrease.

A low temperature suppresses the growth and oxygen consumption rate of Chinhai spiny newt larvae. Such suppression was consistent with the patterns in the thermal acclimatization characteristics of ectotherms [69]. For example, a low temperature could delay the larval development of the relict leopard frog (*Lithobates onca*) [70] and could reduce the basal metabolic rates of Atlantic salmon (*Salmo salar*) [71]. This reflects a type of adaptation strategy involving the suppression of growth and metabolic rate. The reduced body size and head width of larvae at low temperatures may affect their predation ability and conspecific competition, thus reducing energy storage [72]. Accordingly, amphibians usually reduce their energy expenditure by lowering their basal metabolic rates [73]. Our experimental observations revealed an increased incidence of fungal infections (e.g., saprolegniasis [74,75]) in the low-temperature groups [40], which correlated with increased larval mortality. The temperature-driven proliferation of saprolegniasis pathogens may confound larval response metrics. We are currently implementing pathogen load quantification and antifungal protocols to isolate direct thermal impacts. It needs to be clearly pointed out that all acclimation groups were maintained with the same water source in order to eliminate the confounding effects of water quality. Low-temperature acclimation significantly increased locomotor performance in Chinhai spiny newt larvae. A reduced enzyme activity and slowed biochemical reactions under low temperatures collectively decrease metabolic rates [76]. This metabolic suppression allows for a sustained low-intensity locomotion through efficient energy allocation [77]. Cold-acclimated fishes exhibit an improved locomotor performance, which facilitates effective foraging and predator avoidance while maintaining energy balance in low-temperature habitats [78]. Therefore, the energy-efficient mechanisms supporting locomotor performance at low temperatures are not merely physiological traits but represent a crucial ecological adaptation. This allows Chinhai spiny newt larvae to maintain essential foraging efficiency and predator avoidance capabilities across a broader thermal range or during thermally challenging periods (e.g., cool mornings, evenings, or seasonal transitions). This expanded thermal niche for critical activities could significantly contribute to individual survival and reproductive success within their habitat.

**Figure 6:**
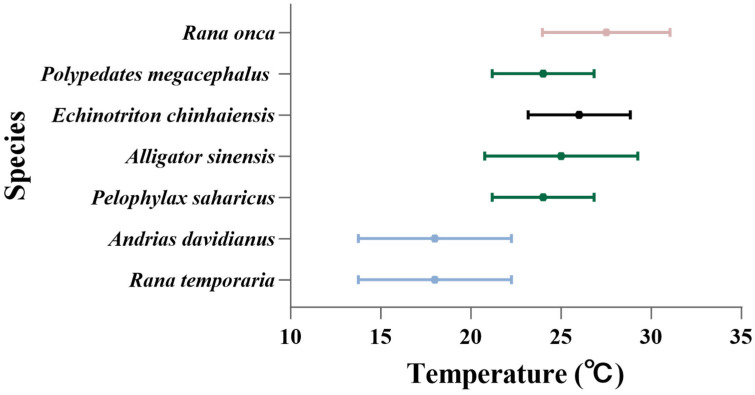
Optimal temperature range for seven amphibians and reptiles.

The optimal temperatures range varies between species and groups (Figure 6). Chinhai spiny newt larvae acclimation at 24 °C and 28 °C exhibited larger body sizes and higher oxygen consumption rates compared to the 20 °C and 32 °C acclimation groups. This thermal response delineates 24–28 °C to be the optimal temperature range for this species. There is a great spatial distributional specificity in the vulnerability of ectotherms to climate change [70,79,80,81,82,83,84]. Compared with other ectotherms, Chinhai spiny newt larvae in the subtropics have a higher optimal temperature range and temperature threshold compared to temperate amphibians (e.g., *Rana temporaria*), but a lower one than tropical amphibians (e.g., *Polypedates megacephalus*) (Figure 6). Their thermodynamic traits are similar to those of other subtropical reptiles, such as the Chinese alligator (*Alligator sinensis*) (Figure 6). Moreover, the thermal tolerance of many organisms is proportional to the magnitude of temperature change they experience; this climatic feature also increases with latitude [85,86]. Tropical species demonstrate a particular vulnerability due to their narrow thermal safety margins, as current environmental temperatures already approximate their physiological optima [87]. Conversely, high-latitude species exhibit a broader thermal plasticity and inhabit environments below their optimal temperature ranges. These traits may allow them to benefit from short-term warming [66]. The Chinhai spiny newt, which is a subtropical species, thrives in an optimal temperature range of 24–28 °C; this range is limited in ecological applicability due to the results, which are based on the constant temperature design. In wild habitats, Chinhai spiny newt experience diel and seasonal temperature fluctuations. However, due to the fact that the monitoring equipment of still-water ponds in the habitats of Chinhai spiny newts is incomplete, the pattern of diurnal and seasonal temperature change in still-water ponds is still unclear. However, based on our field surveys (from May to August in 2024), the water temperatures of still-water ponds are between 22 and 27 °C [40], which overlaps with the range of 24–28 °C. In order to compare the optimal temperature range and habitat temperature ranges, as well as to make conservation decisions to cope with global warming, the long-term temperature monitoring of habitats should be strengthened in the future.

High temperatures suppress the growth, oxygen consumption rate, and locomotor performance of Chinhai spiny newt larvae, which delineate a critical temperature threshold for physiological trade-offs. This thermal stress response is also verified across ectotherms. Axolotl (*Ambystoma mexicanum*) acclimated at 27 °C exhibit a reduced body weight and elevated mortality rates. Under high temperatures, larvae may use more energy to maintain their basal metabolism and heat stress response, thus limiting the allocation of resources for growth [44,73]. The reduced body size and head width of the larvae may affect their predation ability, thereby constraining growth and development due to limited energy acquisition [72]. Comparable thermo-metabolic constraints occur in other amphibians; for example, the black-spotted pond frog (*Pelophylax nigromaculatus*) shows marked oxygen consumption declines at temperatures above 28 °C [88]. Additionally, African clawed frog tadpoles exhibit depressed mitochondrial respiration rates and a reduced motility after acclimation at 30 °C [89]. This metabolic depression likely stems from heat-induced mitochondrial dysfunction and enzymatic inactivation [90]. High temperatures impair larval locomotor performance through dual synergistic pathways. On the one hand, high temperatures reduce oxidative damage through metabolic inhibition, but they simultaneously lead to an insufficient energy supply [91]. On the other hand, high temperatures alter muscle structure and limit oxygen transport, directly impairing larval locomotor performance [92].

The experimental temperatures had significant effects on the oxygen consumption rate and locomotor performance of Chinhai spiny newt larvae. This suggests that larvae are more sensitive to acute temperature changes compared to slow temperature changes. Both alpine (*Ichthyosaura alpestris*) and smooth (*Lissotriton vulgaris*) newts were significantly affected by acute temperature changes [11], which were unable to reduce the thermal sensitivity of physiological traits in order to buffer energetic demands [93]. Therefore, climate change-induced weather extremes seriously threaten the survival of Chinhai spiny newt larvae. The “beneficial acclimation hypothesis” posits that prolonged environmental conditioning enhances organismal fitness within corresponding habitats [10]. While acclimation at 20 °C and 32 °C enhanced locomotor performance at matching experimental temperatures (supporting the “beneficial acclimation hypothesis”), this pattern weakened at 24–28 °C. This result was different from that of the oriental fire-bellied newt (*Cynops orientalis*) acclimated at different temperatures, whose locomotor performance was measured at different experimental temperatures; the results supported the beneficial acclimation hypothesis [94]. In many insect studies, the findings do not support the “beneficial acclimation hypothesis”. This may be due to the influence of phenotypic plasticity on the results of the study, as well as the unreasonable setting of the acclimation temperature and the time of acclimation [95].

### Limitations

It should be noted that the temperature control equipment used in this study needs to be updated, which lead to two limitations. On the one hand, the experimental design tested four discrete temperatures (20 °C, 24 °C, 28 °C, and 32 °C) at 4 °C intervals. This is a simple design that is not able to capture the more precise temperature variations within the range of 24 °C to 28 °C (e.g., 26 °C). Consequently, this optimal range is a preliminary estimate based on the existing data points. On the other hand, while our study employed a constant temperature design to investigate the effects of specific temperatures on physiological performance, this approach inherently simplifies the complexity of natural thermal environments [96]. Studies with static temperatures, though methodologically essential for isolating specific thermal responses, risk overestimating or underestimating climate [46]. However, the study conducted in the constant temperature design is basic, and should be used to supplement the fluctuating temperature design in order to predict climate change impacts. Future studies should be based on controlled fluctuation temperature equipment (e.g., semi-natural climate chambers [46]) in order to explore the more precise optimal temperature range and thermal biology adaption of this threatened species.

## 5. Conclusions 

By measuring the growth indices, basal metabolic rate, and locomotor performance of Chinhai spiny newt larvae, we concluded that (1) the optimal temperature range was between 24 and 28 °C; (2) low temperatures inhibited the growth and basal metabolic rate of Chinhai spiny newt larvae, but enhanced locomotor performance, while high temperatures inhibited larval growth, basal metabolic rate, and locomotor performance, and the temperature threshold was 32 °C; and (3) acute temperature changes significantly affected the basal metabolic rate and locomotor performance of the larvae.

On account of these findings, we recommend that (1) the implementation of artificial breeding should be based on the optimal temperature range in order to improve the survival rate of Chinhai spiny newt larvae; (2) a temperature monitoring system should be established to ensure the linkage to early warming (such as when continuous temperatures are close to or exceed 32 °C, whereby slow, small-scale water addition or replacement could be immediately activated); and (3) a temperature threshold should be integrated into the species distribution model in order to predict an accurate and suitable habitat under conditions of climate change. While this study examined the effects of constant temperatures on the morphology and metabolism of Chinhai spiny newt larvae, additional research should focus on the consequences of variable thermal environments.

## Figures and Tables

**Table 1:** Summary of ANOVA examining the morphometric parameters of Chinhai spiny newt larvae among acclimation temperatures, acclimation times, and the interaction of acclimation temperatures and acclimation times. Significant differences (*p* < 0.05) between different groups are highlighted in bold.

Morphometric Parameters	Factor	Num df	Den df	*F* Value	*p*
Body weight	**Acclimation temperature**	**3**	**18.510**	**69.642**	**<0.001**
(g)	**Acclimation time**	**6**	**1435.250**	**1526.547**	**<0.001**
	**Acclimation temperature** × **Acclimation time**	**18**	**1434.320**	**14.345**	**<0.001**
Snout–vent length	**Acclimation temperature**	**3**	**23.800**	**104.452**	**<0.001**
(cm)	**Acclimation time**	**6**	**1435.000**	**1447.040**	**<0.001**
	**Acclimation temperature** × **Acclimation time**	**18**	**1434.500**	**10.778**	**<0.001**
Head length	**Acclimation temperature**	**3**	**15.670**	**43.573**	**<0.001**
(cm)	**Acclimation time**	**6**	**1432.560**	**647.221**	**<0.001**
	**Acclimation temperature** × **Acclimation time**	**18**	**1430.880**	**4.868**	**<0.001**
Head width	**Acclimation temperature**	**3**	**12.890**	**88.056**	**<0.001**
(cm)	**Acclimation time**	**6**	**1427.210**	**1105.180**	**<0.001**
	**Acclimation temperature** × **Acclimation time**	**18**	**1425.790**	**11.370**	**<0.001**

**Table 2:** The effects of acclimation temperatures, experimental temperatures, and their interaction on the basal metabolic rate of Chinhai spiny newt larvae, shown as type III analysis-of-deviance tables. Significant differences (*p* < 0.05) between different groups are highlighted in bold.

Basal Metabolic Rate	X^2^	*df*	*p*
Acclimation temperature	4.955	3	0.175
**Experimental temperature**	**8.650**	**3**	**0.034**
Acclimation temperature × Experimental temperature	5.919	9	0.748

**Table 3:** The effects of acclimation temperatures, experimental temperatures, and their interaction on the locomotor performance of Chinhai spiny newt larvae, shown as type III analysis-of-deviance tables. Significant differences (*p* < 0.05) between different groups are highlighted in bold.

Locomotor Performance	Chisq	*df*	*p*
**Acclimation temperature**	**42.312**	**3**	**<0.001**
**Experimental temperature**	**31.683**	**3**	**<0.001**
**Acclimation temperature** × **Experimental temperature**	**44.660**	**9**	**<0.001**

## Data Availability

Data will be made available on demand.

## Supplementary Materials

The following supporting information can be downloaded at: https://www.mdpi.com/article/10.3390/biology14080942/s1, Figure S1. Water temperature in still-water ponds of wild Chinhai spiny newt larval habitats (May–July). Figure S2. Normality tests of morphometric parameter models under different acclimation temperature conditions. (a) Body weight (BW), (b) snout–vent length (SVL), (c) head length (HL), and (d) head width (HW). Figure S3. Histograms and density curves of oxygen consumption rate distributions in different experimental temperatures. (a) The experimental temperature was 20 °C. (b) The experimental temperature was 24 °C. (c) The experimental temperature was 28 °C. (d) The experimental temperature was 32 °C. (e) Measured at acclimation temperature. Figure S4. Residual diagnostics of morphometric parameter models under different acclimation temperature conditions. (a) Body weight (BW), (b) snout–vent length (SVL), (c) head length (HL), and (d) head width (HW). Table S1. Dispersion analysis of locomotor performance in different experimental temperatures—mean, variance, and ratio. Table S2. Summary of linear mixed models to examine the differences in the body weight of Chinhai spiny newt larvae under different acclimation temperatures. Significant differences (*p* < 0.05) between different groups are highlighted in bold. Table S3. Post hoc pairwise comparisons of larval body weight across acclimation temperatures at each acclimation time. Significant differences (*p* < 0.05) between different groups are highlighted in bold. Table S4. Summary of linear mixed models to examine the differences in the snout–vent length of Chinhai spiny newt larvae under different acclimation temperatures. Significant differences (*p* < 0.05) between different groups are highlighted in bold. Table S5. Post hoc pairwise comparisons of larval snout–vent length across acclimation temperatures at each acclimation time. Significant differences (*p* < 0.05) between different groups are highlighted in bold. Table S6. Summary of linear mixed models to examine the differences in the head length of Chinhai spiny newt larvae under different acclimation temperatures. Significant differences (*p* < 0.05) between different groups are highlighted in bold. Table S7. Post hoc pairwise comparisons of larval head length across acclimation temperatures at each acclimation time. Significant differences (*p* < 0.05) between different groups are highlighted in bold. Table S8. Summary of linear mixed models to examine the differences in the head width of Chinhai spiny newt larvae under different acclimation temperatures. Significant differences (*p* < 0.05) between different groups are highlighted in bold. Table S9. Post hoc pairwise comparisons of larval head width across acclimation temperatures at each acclimation time. Significant differences (*p* < 0.05) between different groups are highlighted in bold. Table S10. Residual diagnostics of oxygen consumption rate models under different experimental temperature conditions using DHARMa. Table S11. Residual diagnostics of the interaction of acclimation and experimental temperatures on the oxygen consumption rate model using DHARMa. Table S12. Summary of generalized linear mixed models to examine the differences in the oxygen consumption rate of Chinhai spiny newt larvae under different temperatures. Significant differences (*p* < 0.05) between different groups are highlighted in bold. Table S13. Summary of generalized linear mixed models to investigate the differences in oxygen consumption rates of Chinhai spiny newt larvae between acclimation and control groups under different experimental temperatures. Significant differences (*p* < 0.05) between groups are marked in bold. Table S14. Results of Dunn’s post hoc test following Kruskal–Wallis analysis of oxygen consumption rates among temperature groups at the 28 °C experimental condition. Significant differences (*p* < 0.05) between groups are marked in bold. Table S15. Residual diagnostics of locomotor performance models under different experimental temperature conditions using DHARMa. Table S16. Residual diagnostics of the interaction of acclimation and experimental temperatures on the locomotor performance model using DHARMa. Table S17. Summary of generalized linear mixed models to examine the differences in the locomotor performance of Chinhai spiny newt larvae under different temperatures. Significant differences (*p* < 0.05) between different groups are highlighted in bold. Table S18. Summary of generalized linear mixed models to investigate the differences in locomotor performance of Chinhai spiny newt larvae between acclimation and control groups under different experimental temperatures. Significant differences (*p* < 0.05) between groups are marked in bold.
