# Supplementary material for: High Warming Restricts the Growth and Movement of a Larval Chinese Critically Endangered Relict Newt
Source: Biology (Basel). 2025 Jul 27;14(8):942. doi: 10.3390/biology14080942 (PMC12383594; doi:10.3390/biology14080942)
Supplement: Supplementary file 1 [file biology-14-00942-s001.zip › biology-3779637-supplementary.pdf]

Supplemental material

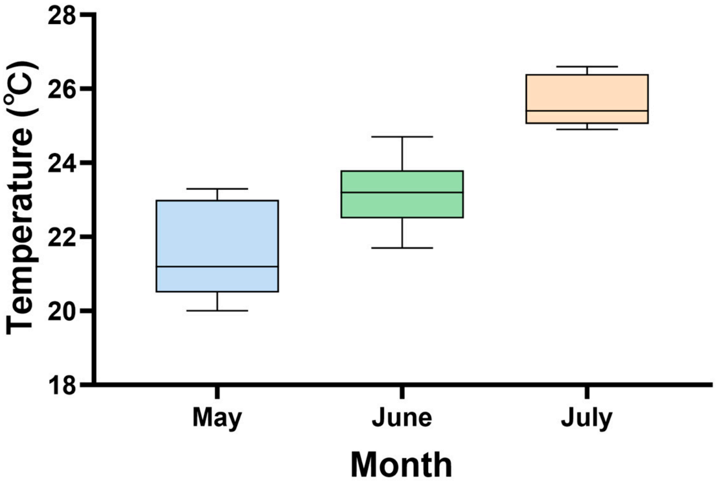

Figure S1. Water temperature in still-water ponds of wild Chinhai spiny newt larval habitats (May–July).

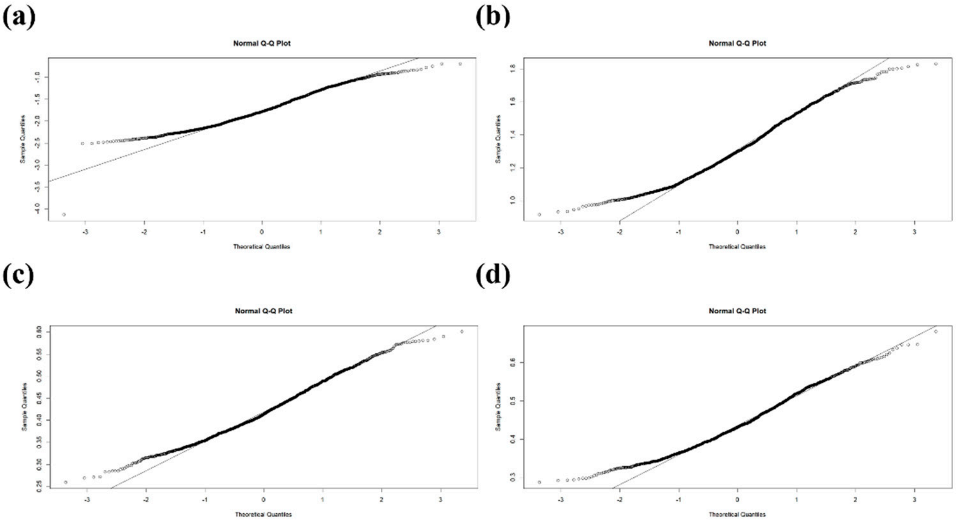

Figure S2. Normality tests of morphometric parameter models under different acclimation temperature conditions. (a) body weight (BW), (b) snout–vent length (SVL), (c) head length (HL), and (d) head width (HW)

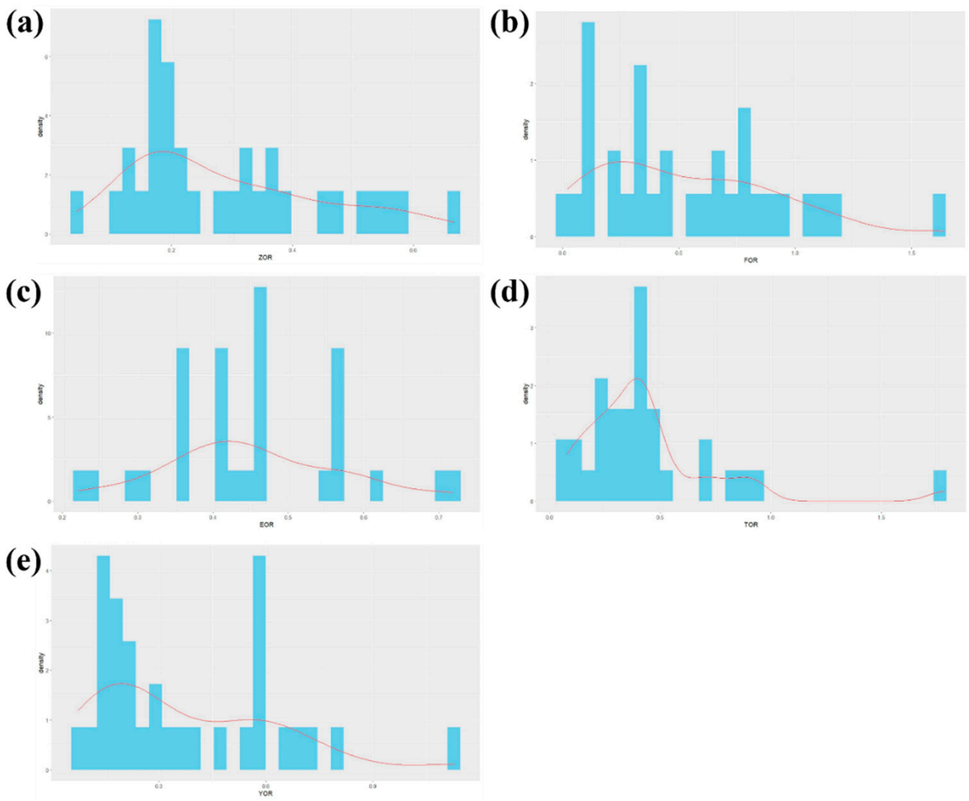

Figure S3. Histograms and density curves of oxygen consumption rate distributions in different experimental temperatures. (a) The experimental temperature was 20°C. (b) The experimental temperature was 24°C. (c) The experimental temperature was 28°C. (d) The experimental temperature was 32°C. (e) Measured at acclimation temperature.

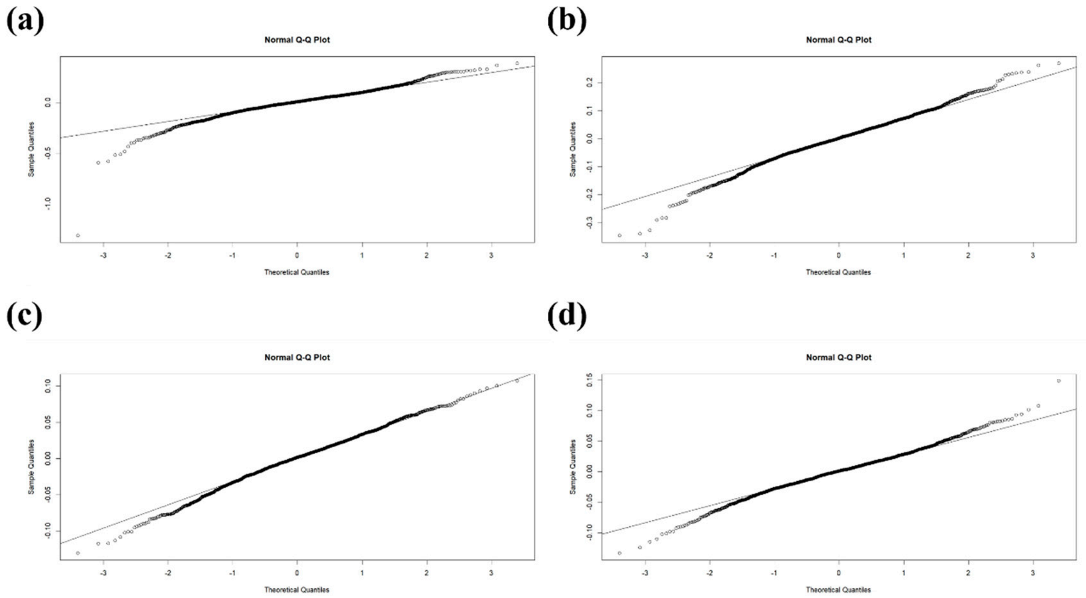

Figure S4. Residual diagnostics of morphometric parameter models under different acclimation temperature conditions. (a) Body weight (BW), (b) snout–vent length (SVL), (c) head length (HL), and (d) head width (HW).

Table S1. Dispersion analysis of locomotor performance in different experimental temperatures—mean, variance, and ratio.

Experimental temperature	Mean	Variance	Ratio
24 (°C)	42.54	875.59	20.58
20 (°C)	54.09	1412.64	26.11
28 (°C)	60.37	818.14	13.55
32 (°C)	40.70	495.44	12.17
Acclimation temperature (°C)	54.80	1210.38	22.09

Table S2. Summary of linear mixed models to examine the differences in the body weight of Chinhai spiny newt larvae under different acclimation temperatures. Significant differences ($p < 0.05$) between different groups are highlighted in bold.

Acclimation Time (day)	Groups (treatment temperature VS control)	Estimate	Standard error	*t* value	*p* value
4	20°C VS 24°C	-0.041	0.033	-1.241	0.215
	28°C VS 24°C	0.010	0.032	0.332	0.740
	32°C VS 24°C	-0.015	0.032	-0.454	0.650
8	**20°C VS 24°C**	**-0.101**	**0.034**	**-2.937**	**0.003**
	28°C VS 24°C	0.014	0.034	0.412	0.680
	32°C VS 24°C	-0.053	0.034	-1.566	0.117
11	**20°C VS 24°C**	**-0.171**	**0.034**	**-4.945**	**<0.001**
	28°C VS 24°C	0.043	0.034	1.273	0.203
	**32°C VS 24°C**	**-0.139**	**0.034**	**-4.120**	**<0.001**
14	**20°C VS 24°C**	**-0.219**	**0.035**	**-6.270**	**<0.001**
	28°C VS 24°C	0.028	0.034	0.831	0.406
	**32°C VS 24°C**	**-0.213**	**0.035**	**-6.296**	**<0.001**
18	**20°C VS 24°C**	**-0.224**	**0.037**	**-5.985**	**<0.001**
	28°C VS 24°C	0.047	0.034	1.367	0.171
	**32°C VS 24°C**	**-0.212**	**0.034**	**-6.224**	**<0.001**

Table S3. Post hoc pairwise comparisons of larval body weight across acclimation temperatures at each acclimation time. Significant differences ($p < 0.05$) between different groups are highlighted in bold.

**Acclimation Time (day)**	**Groups (treatment temperature VS control)**	**Estimate**	**Standard error**	***t* value**	***p* value**
4	**20°C VS 24°C**	**0.084**	**0.032**	**2.579**	**0.026**
	28°C VS 24°C	-0.016	0.032	-0.498	0.746
	32°C VS 24°C	-0.021	0.033	-0.652	0.746
8	**20°C VS 24°C**	**0.144**	**0.034**	**4.255**	**<0.001**
	28°C VS 24°C	-0.019	0.034	-0.572	0.623
	32°C VS 24°C	0.017	0.034	0.495	0.623
11	**20°C VS 24°C**	**0.213**	**0.034**	**6.305**	**<0.001**
	28°C VS 24°C	-0.048	0.034	-1.434	0.158
	**32°C VS 24°C**	**0.103**	**0.034**	**3.033**	**0.005**
14	**20°C VS 24°C**	**0.262**	**0.034**	**7.620**	**<0.001**
	28°C VS 24°C	-0.033	0.034	-0.990	0.327
	**32°C VS 24°C**	**0.177**	**0.034**	**5.203**	**<0.001**
18	**20°C VS 24°C**	**0.267**	**0.037**	**7.227**	**<0.001**
	28°C VS 24°C	-0.052	0.034	-1.526	0.133
	**32°C VS 24°C**	**0.034**	**0.034**	**5.139**	**<0.001**

Table S4. Summary of linear mixed models to examine the differences in the snout–vent length of Chinhai spiny newt larvae under different acclimation temperatures. Significant differences (p < 0.05) between different groups are highlighted in bold.

**Acclimation Time (day)**	**Groups (treatment temperature VS control)**	**Estimate**	**Standard error**	***t* value**	***p* value**
4	20°C VS 24°C	-0.010	0.021	-0.482	0.630
	28°C VS 24°C	0.029	0.021	1.389	0.165
	32°C VS 24°C	0.025	0.021	1.182	0.237
8	20°C VS 24°C	-0.026	0.022	-1.156	0.247
	28°C VS 24°C	0.021	0.022	0.955	0.340
	32°C VS 24°C	-0.007	0.022	-0.315	0.753
11	**20°C VS 24°C**	**-0.092**	**0.022**	**-4.155**	**<0.001**
	28°C VS 24°C	0.030	0.022	1.351	0.177
	**32°C VS 24°C**	**-0.064**	**0.022**	**-2.987**	**0.003**
14	**20°C VS 24°C**	**-0.075**	**0.022**	**-3.326**	**<0.001**
	28°C VS 24°C	0.025	0.022	1.162	0.246
	**32°C VS 24°C**	**-0.071**	**0.022**	**-3.286**	**0.001**
18	**20°C VS 24°C**	**-0.110**	**0.024**	**-4.581**	**<0.001**
	28°C VS 24°C	0.031	0.022	1.418	0.156
	**32°C VS 24°C**	**-0.122**	**0.022**	**-5.596**	**<0.001**

Table S5. Post hoc pairwise comparisons of larval snout–vent length across acclimation temperatures at each acclimation time. Significant differences (p < 0.05) between different groups are highlighted in bold.

Acclimation Time (day)	Groups (treatment temperature VS control)	Estimate	Standard error	*t* value	*p* value
4	**20°C VS 24°C**	**0.098**	**0.026**	**3.797**	**0.002**
	28°C VS 24°C	-0.031	0.026	-1.185	0.370
	32°C VS 24°C	-0.026	0.026	-1.010	0.386
8	**20°C VS 24°C**	**0.113**	**0.027**	**4.273**	**<0.001**
	28°C VS 24°C	-0.023	0.027	-0.846	0.486
	32°C VS 24°C	0.005	0.027	0.182	0.857
11	**20°C VS 24°C**	**0.179**	**0.027**	**6.778**	**<0.001**
	28°C VS 24°C	-0.031	0.027	-1.167	0.253
	**32°C VS 24°C**	**0.063**	**0.027**	**2.331**	**0.033**
14	**20°C VS 24°C**	**0.162**	**0.027**	**6.054**	**<0.001**
	28°C VS 24°C	-0.027	0.027	-1.015	0.318
	**32°C VS 24°C**	**0.069**	**0.027**	**2.578**	**0.018**
18	**20°C VS 24°C**	**0.198**	**0.028**	**7.018**	**<0.001**
	28°C VS 24°C	-0.033	0.027	-1.227	0.230
	**32°C VS 24°C**	**0.120**	**0.027**	**4.451**	**<0.001**

Table S6. Summary of linear mixed models to examine the differences in the head length of Chinhai spiny newt larvae under different acclimation temperatures. Significant differences ($p < 0.05$) between different groups are highlighted in bold.

Acclimation Time (day)	Groups (treatment temperature VS control)	Estimate	Standard error	*t* value	*p* value
4	20°C VS 24°C	-0.002	0.009	-0.199	0.842
	28°C VS 24°C	-0.005	0.009	-0.539	0.590
	32°C VS 24°C	0.008	0.009	0.904	0.366
8	20°C VS 24°C	-0.004	0.010	-0.401	0.688
	28°C VS 24°C	-0.012	0.009	-1.299	0.194
	32°C VS 24°C	-0.010	0.009	-1.093	0.274
11	**20°C VS 24°C**	**-0.026**	**0.010**	**-2.691**	**0.007**
	28°C VS 24°C	0.001	0.009	0.151	0.880
	32°C VS 24°C	-0.008	0.010	-0.802	0.423
14	**20°C VS 24°C**	**-0.024**	**0.010**	**-2.451**	**0.014**
	28°C VS 24°C	-0.003	0.009	-0.332	0.740
	**32°C VS 24°C**	**-0.024**	**0.009**	**-2.581**	**0.010**
18	**20°C VS 24°C**	**-0.026**	**0.010**	**-2.478**	**0.013**
	28°C VS 24°C	0.012	0.010	1.246	0.213
	32°C VS 24°C	-0.018	0.010	-1.887	0.060

Table S7. Post hoc pairwise comparisons of larval head length across acclimation temperatures at each acclimation time. Significant differences ($p < 0.05$) between different groups are highlighted in bold.

Acclimation Time (day)	Groups (treatment temperature VS control)	Estimate	Standard error	*t* value	*p* value
4	**20°C VS 24°C**	**0.019**	**0.008**	**2.447**	**0.032**
	28°C VS 24°C	-0.003	0.008	-0.404	0.824
	32°C VS 24°C	-0.003	0.008	-0.416	0.824
8	20°C VS 24°C	0.021	0.008	2.563	0.069
	28°C VS 24°C	0.004	0.008	0.534	0.594
	32°C VS 24°C	0.015	0.008	1.917	0.116
11	**20°C VS 24°C**	**0.043**	**0.008**	**5.230**	**<0.001**
	28°C VS 24°C	-0.009	0.008	-1.168	0.245
	32°C VS 24°C	0.013	0.008	1.563	0.145
14	**20°C VS 24°C**	**0.041**	**0.008**	**4.905**	**<0.001**
	28°C VS 24°C	-0.005	0.008	-0.600	0.550
	**32°C VS 24°C**	**0.030**	**0.008**	**3.656**	**<0.001**
18	**20°C VS 24°C**	**0.043**	**0.009**	**4.709**	**<0.001**
	**28°C VS 24°C**	**-0.020**	**0.008**	**-2.443**	**0.019**
	**32°C VS 24°C**	**0.023**	**0.008**	**2.829**	**0.008**

Table S8. Summary of linear mixed models to examine the differences in the head width of Chinhai spiny newt larvae under different acclimation temperatures. Significant differences ($p < 0.05$) between different groups are highlighted in bold.

Acclimation Time (day)	Groups (treatment temperature VS control)	Estimate	Standard error	*t* value	*p* value
4	20°C VS 24°C	-0.009	0.008	-1.097	0.273
	28°C VS 24°C	0.009	0.008	1.044	0.297
	32°C VS 24°C	0.005	0.008	0.598	0.550
8	**20°C VS 24°C**	**-0.026**	**0.009**	**-2.957**	**0.003**
	28°C VS 24°C	0.002	0.009	0.182	0.855
	32°C VS 24°C	-0.006	0.009	-0.746	0.456
11	**20°C VS 24°C**	**-0.045**	**0.009**	**-5.092**	**<0.001**
	28°C VS 24°C	0.008	0.009	0.927	0.354
	32°C VS 24°C	-0.016	0.009	-1.877	0.060
14	**20°C VS 24°C**	**-0.051**	**0.009**	**-5.687**	**<0.001**
	28°C VS 24°C	0.006	0.009	0.670	0.503
	**32°C VS 24°C**	**-0.037**	**0.009**	**-4.321**	**<0.001**
18	**20°C VS 24°C**	**-0.060**	**0.010**	**-6.289**	**<0.001**
	28°C VS 24°C	0.009	0.009	1.048	0.295
	**32°C VS 24°C**	**-0.036**	**0.009**	**-4.153**	**<0.001**

Table S9. Post hoc pairwise comparisons of larval head width across acclimation temperatures at each acclimation time. Significant differences (p < 0.05) between different groups are highlighted in bold.

Acclimation Time (day)	Groups (treatment temperature VS control)	Estimate	Standard error	*t* value	*p* value
4	**20°C VS 24°C**	**0.023**	**0.008**	**2.816**	**0.014**
	28°C VS 24°C	-0.012	0.008	-1.474	0.177
	**32°C VS 24°C**	**-0.018**	**0.008**	**-2.210**	**0.048**
8	**20°C VS 24°C**	**0.039**	**0.008**	**4.699**	**<0.001**
	28°C VS 24°C	-0.005	0.008	-0.562	0.692
	32°C VS 24°C	-0.006	0.008	-0.762	0.675
11	**20°C VS 24°C**	**0.059**	**0.008**	**6.930**	**<0.001**
	28°C VS 24°C	-0.011	0.008	-1.331	0.227
	32°C VS 24°C	0.004	0.008	0.416	0.679
14	**20°C VS 24°C**	**0.064**	**0.009**	**7.513**	**<0.001**
	28°C VS 24°C	-0.009	0.008	-1.065	0.292
	**32°C VS 24°C**	**0.025**	**0.008**	**2.928**	**0.006**
18	**20°C VS 24°C**	**0.074**	**0.009**	**7.999**	**<0.001**
	28°C VS 24°C	-0.012	0.008	-1.453	0.152
	**32°C VS 24°C**	**0.023**	**0.008**	**2.764**	**0.009**

Table S10. Residual diagnostics of oxygen consumption rate models under different experimental temperature conditions using DHARMa.

Experimental temperature	Test	*p*
20 (°C)	KS test	0.998
	Dispersion test	0.928
	Outlier test	0.226
	Levene test	>0.050
	Nonparametric dispersion test	0.928
24 (°C)	KS test	0.457
	Dispersion test	0.784
	Outlier test	1.000
	Levene test	>0.050
	Nonparametric dispersion test	0.784
28 (°C)	KS test	0.002
	Dispersion test	0.880
	Outlier test	0.226
	Levene test	<0.050
	Nonparametric dispersion test	0.880
32 (°C)	KS test	0.730
	Dispersion test	0.424
	Outlier test	1.000
	Levene test	>0.050
	Nonparametric dispersion test	0.424
Acclimation temperature (°C)	KS test	0.989
	Dispersion test	0.648
	Outlier test	1.000
	Levene test	>0.050
	Nonparametric dispersion test	0.648

Table S11. Residual diagnostics of the interaction of acclimation and experimental temperatures on the oxygen consumption rate model using DHARMa.

Factor	Test	*p*
Acclimation temperature × Experimental temperature	KS test	0.561
	Dispersion test	0.568
	Outlier test	0.571
	Levene test	>0.050
	Nonparametric dispersion test	0.568

Table S12. Summary of generalized linear mixed models to examine the differences in the oxygen consumption rate of Chinhai spiny newt larvae under different temperatures. Significant differences ($p < 0.05$) between different groups are highlighted in bold.

Groups	Estimate	Standard error	*z* value	*p* value
**20°C VS 24°C**	**-0.675**	**0.319**	**-2.118**	**0.034**
28°C VS 24°C	0.307	0.311	0.987	0.324
32°C VS 24°C	-0.444	0.319	-1.393	0.164

Table S13. Summary of generalized linear mixed models to investigate the differences in oxygen consumption rates of Chinhai spiny newt larvae between acclimation and control groups under different experimental temperatures. Significant differences ($p < 0.05$) between groups are marked in bold.

Groups	Acclimation temperature VS experimental temperature	Estimate	Standard error	*z* value	*p* value
20°C	**20°C VS 24°C**	**0.612**	**0.249**	**2.456**	**0.014**
	20°C VS 28°C	0.341	0.249	1.368	0.171
	20°C VS 32°C	0.149	0.249	0.596	0.551
24°C	24°C VS 20°C	0.114	0.379	0.302	0.763
	24°C VS 28°C	0.593	0.379	1.566	0.117
	24°C VS 32°C	0.018	0.379	0.047	0.962
28°C	**28°C VS 20°C**	**-0.346**	**0.100**	**-3.454**	**0.001**
	**28°C VS 24°C**	**-0.209**	**0.100**	**-2.085**	**0.037**
	**28°C VS 32°C**	**-0.443**	**0.100**	**-4.419**	**<0.001**
32°C	32°C VS 20°C	0.305	0.242	1.259	0.208
	**32°C VS 24°C**	**0.689**	**0.245**	**2.811**	**0.005**
	**32°C VS 28°C**	**0.618**	**0.245**	**2.523**	**0.012**

Table S14. Results of Dunn's post hoc test following Kruskal–Wallis analysis of oxygen consumption rates among temperature groups at the 28°C experimental condition. Significant differences ($p < 0.05$) between groups are marked in bold.

Groups	Acclimation temperature VS experimental temperature	*z*	*p* value
28°C	**28°C VS 20°C**	**-2.765**	**0.017**
	28°C VS 24°C	-1.074	0.339
	**28°C VS 32°C**	**3.571**	**0.002**

Table S15 Residual diagnostics of locomotor performance models under different experimental temperature conditions using DHARMa.

Experimental temperature	Test	*p*
20 (°C)	KS test	0.841
	Dispersion test	0.232
	Outlier test	1.000
	Levene test	>0.050
	Nonparametric dispersion test	0.232
24 (°C)	KS test	0.288
	Dispersion test	0.376
	Outlier test	1.000
	Levene test	>0.050
	Nonparametric dispersion test	0.376
28 (°C)	KS test	0.187
	Dispersion test	0.424
	Outlier test	0.840
	Levene test	>0.050
	Nonparametric dispersion test	0.424
32 (°C)	KS test	0.199
	Dispersion test	0.256
	Outlier test	0.480
	Levene test	>0.050
	Nonparametric dispersion test	0.256
Acclimation temperature (°C)	KS test	0.589
	Dispersion test	0.600
	Outlier test	0.580
	Levene test	>0.050
	Nonparametric dispersion test	0.600

Table S16. Residual diagnostics of the interaction of acclimation and experimental temperatures on the locomotor performance model using DHARMa.

Factor	Test	*p*
Acclimation temperature × Experimental temperature	KS test	0.557
	Dispersion test	0.232
	Outlier test	0.980
	Levene test	>0.050
	Nonparametric dispersion test	0.232

Table S17. Summary of generalized linear mixed models to examine the differences in the locomotor performance of Chinhai spiny newt larvae under different temperatures. Significant differences ($p < 0.05$) between different groups are highlighted in bold.

Groups	Estimate	Standard error	*z* value	*p* value
**20°C VS 24°C**	**-0.443**	**0.124**	**-3.560**	**<0.001**
28°C VS 24°C	-0.002	0.136	-0.020	0.988
32°C VS 24°C	-0.062	0.136	-0.450	0.651

Table S18. Summary of generalized linear mixed models to investigate the differences in locomotor performance of Chinhai spiny newt larvae between acclimation and control groups under different experimental temperatures. Significant differences ($p < 0.05$) between groups are marked in bold.

Groups	Treatment temperature VS experimental temperature	Estimate	Standard error	*z* value	*p* value
20°C	20°C VS 24°C	0.206	0.158	1.310	0.190
	20°C VS 28°C	0.291	0.157	1.850	0.065
	30°C VS 32°C	0.168	0.158	1060	0.288
24°C	**24°C VS 20°C**	**-0.327**	**0.146**	**-2.250**	**0.025**
	24°C VS 28°C	0.257	0.144	1.790	0.074
	24°C VS 32°C	0.039	0.146	0.270	0.785
28°C	28°C VS 20°C	-0.177	0.110	-1.610	0.108
	28°C VS 24°C	0.055	0.126	0.440	0.660
	28°C VS 32°C	0.066	0.094	0.700	0.485
32°C	32°C VS 20°C	0.012	0.122	0.100	0.920
	32°C VS 24°C	0.190	0.121	1.570	0.117
	**32°C VS 28°C**	**0.272**	**0.121**	**2.240**	**0.025**
